# Burgeoning Exploration of the Role of Natural Killer Cells in Anti-PD-1/PD-L1 Therapy

**DOI:** 10.3389/fimmu.2022.886931

**Published:** 2022-05-12

**Authors:** Rilan Bai, Jiuwei Cui

**Affiliations:** Cancer Center, The First Hospital of Jilin University, Changchun, China

**Keywords:** tumor, immune checkpoint inhibitor, natural killer cell, programmed death receptor-1, programmed death-ligand 1

## Abstract

Antibodies targeting programmed death receptor-1 (PD-1)/programmed death ligand-1 (PD-L1) have been considered breakthrough therapies for a variety of solid and hematological malignancies. Although cytotoxic T cells play an important antitumor role during checkpoint blockade, they still show a potential killing effect on tumor types showing loss of/low major histocompatibility complex (MHC) expression and/or low neoantigen load; this knowledge has shifted the focus of researchers toward mechanisms of action other than T cell-driven immune responses. Evidence suggests that the blockade of the PD-1/PD-L1 axis may also improve natural killer (NK)-cell function and activity through direct or indirect mechanisms, which enhances antitumor cytotoxic effects; although important, this topic has been neglected in previous studies. Recently, some studies have reported evidence of PD-1 and PD-L1 expression in human NK cells, performed exploration of the intrinsic mechanism by which PD-1/PD-L1 blockade enhances NK-cell responses, and made some progress. This article summarizes the recent advances regarding the expression of PD-1 and PD-L1 molecules on the surface of NK cells as well as the interaction between anti-PD-1/PD-L1 drugs and NK cells and associated molecular mechanisms in the tumor microenvironment.

## 1 Introduction

Antibodies targeting programmed death receptor-1 (PD-1) and programmed death ligand 1 (PD-L1) have been approved for the treatment of a variety of solid and hematologic malignancies; patients with various malignancies and even those with a very advanced disease showed durable responses to this treatment (1–3). However, only 10–20% of patients with different tumor types respond well to PD-1/PD-L1 blocking therapy (3, 4). In addition, treatment with anti-PD-1/PD-L1 monoclonal antibodies (mAbs) can lead to unexplained clinical responses of tumors with no or low expression of major histocompatibility complex (MHC) and/or PD-L1 (2, 4, 5). Therefore, a better understanding of the mechanisms of action of anti-PD-L1 (or anti-PD-1) mAb therapy and the impact of this therapy on each component of the tumor immune microenvironment will help improve the precision of cancer immunotherapeutics in the future. Most believe that antibodies targeting PD-1 and PD-L1 are largely only beneficial for eliciting T cell-driven responses, but accumulating evidence suggests that blocking the PD-1/PD-L1 axis may also improve natural killer (NK)-cell function and activity and enhance antitumor cytotoxicity through direct/indirect but crucial mechanisms. For example, Hodgkin lymphoma cells with defective MHC class I expression responds well to anti-PD-1 mAb therapy, which suggests the presence of immune responses that are independent of cytotoxic CD8^+^T cells, inhibited by PD-1, and rescued by anti-PD-1 therapy (5–7). NK cells show MHC-independent antitumor cytotoxicity, which allows them to exhibit killing effects on many tumor types with absent or low MHC expression and/or low neoantigen burden. Recently, some studies have reported evidence of PD-1 and PD-L1 expression in human NK cells (8, 9). *In vivo* mechanistic studies on whether and how PD-1 or PD-L1 plays a role in NK-cell response to tumors and whether and how PD-1/PD-L1 blockade mobilizes NK cell response remain scarce; however, with the development of multi-omics and high-throughput sequencing technologies, the understanding of this field has gradually deepened and progressed. Based on this knowledge, in this article, we comprehensively review the expression of PD-1 and PD-L1 molecules on the surface of NK cells and discuss the interactions between anti-PD-1/PD-L1 drugs and NK cells in the tumor microenvironment (TME) as well as the associated molecular mechanisms.

## 2 NK Cell Function and Phenotype

NK cells are mainly involved in killing microbes and malignantly transformed allogeneic and autologous cells without prior sensitization and exhibit non-MHC-restricted antitumor cytotoxicity (10). Activated NK cells exert a strong cytotoxic effect by inducing the secretion of cytotoxic mediators and the production of inflammatory cytokines and chemokines through integration of adhesion molecules and receptor signaling activation (11). According to the CD56 density on the cell surface, NK cells can be divided into CD56^dim^ (~90%) and CD56^dim^ (~10%) cells (12). CD56^bright^ mainly performs immunoregulatory function by secreting cytokines, while CD56^dim^ mainly performs cytolytic function by secreting granzyme B and perforin, which enhance the expression of immunoglobulin-like receptors and Fcγ receptor III (FcγRIII)/CD16 (13, 14). Induction and regulation of NK cell function is mediated by a range of activating or inhibitory surface receptors. In humans, the main activating receptors involved in target-cell killing include natural cytotoxic receptors (NCRs) (including NKp46, NKp30, and NKp44) and NKG2D (15). FcγRIII is also an activating receptor that is mainly expressed by CD56^dim^NK cells and is essential for antibody-dependent cell-mediated cytotoxicity (ADCC) against IgG-coated target cells (16). Conversely, MHC class I antigen-specific inhibitory receptors on the surface of NK cells, namely, killer cell immunoglobulin-like receptors (KIRs), leukocyte immunoglobulin-like receptors (LIRs), and natural killer group 2 A (NKG2A), tightly regulate the cytotoxicity mediated by these cells and the production of lymphokines, by recognizing self MHC-I antigens (17, 18). In addition, several non-MHC-specific inhibitory NK receptors have been identified, including classical cytotoxic T-lymphocyte-associated protein 4 (CTLA-4), PD-1, and T cell immunoreceptor with Ig and immunoreceptor tyrosine-based inhibitory motif (ITIM) domains (TIGIT), CD96, lymphocyte-activation gene 3 (LAG-3), and T cell immunoglobulin-3 (TIM-3) (19). Sheffer M et al. (20) systematically defined the molecular signature of human tumor cells that determines their sensitivity to human allogeneic NK cells and found that the transcriptional signature of NK cell-sensitive tumor cells correlates with immune checkpoint inhibitor (ICI) resistance in clinical samples. The study has also applied genome-scale CRISPR-based gene editing screens in several solid tumor cell lines to functionally interrogate which genes in tumor cells regulate responses to NK cells. Tumor cells escape immune responses by regulating the expression of inhibitory receptors on NK cells, and in this context, PD-1/PD-L1 axis has been studied extensively; understanding the expression of NK cell surface receptors and their role in the functional activity of NK cells is essential for the development of effective immunotherapies.

## 3 PD-1/PD-L1 on NK Cells: Expression, Regulation, and Effects on Cell Function

### 3.1 PD-1 on NK Cells: Expression, Regulation, and Effect on Cell Function

PD-1 is a member of the immunoglobulin superfamily. It can be expressed on various immune cells, including T (CD4^+^ and CD8^+^) cells, B cells, bone marrow cells, NK cells, and other innate lymphocytes (ILCs) (21–23). When bound to ligands (PD-L1 and PD-L2) that may be expressed on tumor cells, PD-1 may play a role in impairing antitumor effects and facilitating tumor immune escape (21–23). In recent years, researchers have shown interest in targeting PD-1 to increase NK activity. However, PD-1 expression on NK cells is diverse and difficult to clarify. High expression of PD-1 on NK cells can be detected in the peripheral blood of approximately one-quarter of healthy individuals (22). PD-1, which is usually not expressed on CD56^bright^ NK cells, is confined to fully mature NK cells of NKG2A^−^KIR^+^CD57^+^CD56^dim^ phenotype (24). In the TMEs of various cancers, such as ovarian cancer (ascites), Kaposi’s sarcoma (peripheral blood), renal cell carcinoma, and multiple myeloma, the proportion of PD-1^+^ NK cells and the expression of PD-1 on NK cells increase (25–28). PD-1 expression on peripheral and tumor infiltrating NK cells from patients with digestive cancers was increased (29). In addition, chronic infections such as those caused by human immunodeficiency virus (HIV), hepatitis C virus (HCV), and human cytomegalovirus (HCMV) have also been shown to enhance PD-1 expression on NK cells (30, 31).

PD-1, an important checkpoint of NK activation, is more abundantly expressed in activated NK cells than in inactivated NK cells (9). Functional and phenotypic assays showed that PD-1^+^ NK cells had the highest functional activity when stimulated and that most of these cells expressed activation markers (CD69 and Sca-1) (32). A higher frequency of circulating PD-1^+^ NK cells (mean > 9%) was associated with a better overall survival (OS) in patients with head and neck cancers (HNCs) (33), which indicates that PD-1^+^ NK cells are not necessarily inactive in PD-L1^+^ tumors but may only be inhibited from killing tumor cells. The interaction of PD-1 with its ligands provides a negative signal for the activation of NK cells, which causes the NK cells to express a dysfunctional, exhausted phenotype (26) and an inhibited antitumor ability (29). Several murine tumor models have shown that PD-1^+^ NK cells present at the site of PD-L1^+^ tumors exhibit an exhaustive phenotype and that PD-1/PD-L1 interaction strongly inhibits NK cell-mediated antitumor immunity (32). Overall, PD-1^+^ NK cells exhibit enhanced apoptotic sensitivity, reduced cytolytic activity, impaired cytotoxic and cytokine production efficiencies, and reduced proliferative capacity in PD-L1^+^ tumors (24, 29). In addition, immune cells in TME can also affect NK cells by expressing PD-L1. For example, tumor-associated neutrophils (TANs) can impair the cytotoxicity and infiltration capacity of NK cells, and downregulation of CCR1 leads to diminished infiltration capacity of NK cells and reduced responsiveness of the NK-activating receptors NKp46 and NKG2D (34); CD56^dim^PD-1^+^ NK cells expressed in patients with Hodgkin lymphoma are efficiently inhibited by PD-L1-expressing myeloid cells (35).

The molecular mechanisms regulating PD-1 expression in human NK cells have not yet been elucidated. Signaling by cells and/or soluble factors in the TME may play a major role, and cytokines may mediate crosstalk between different immune checkpoints. For example, it has been demonstrated that tumor-derived interleukin (IL)-18 increases the immunosuppressive CD56^dim^CD16^dim^/^-^ NK cell fraction in patients with triple-negative breast cancer (TNBC) and induces the expression of PD-1 in these cells (36). The G-CSF/STAT3 pathway and IL-18 are responsible for upregulating PD-L1 expression on TANs and NK cells, respectively; transforming growth factor-β (TGF-β) and interferon-γ (IFN-γ) impair NK cell cytotoxicity by upregulating the expressions of PD-L1 and PD-1 on tumor cells and NK cells, respectively (37). In addition, chemotherapeutic agents can upregulate PD-1 expression on NK cells and PD-L1 expression on tumor cells through nuclear factor kappa B (NF-κB) (38). In future studies, it is important to identify the mechanisms that lead to the expression of PD-1 on NK cells in the TME and their importance in NK cell-based immunotherapy.

### 3.2 PD-L1 on NK Cells: Expression, Regulation, and Effect on Cell Function

IFN-γ secreted by activated T cells can stimulate the upregulation of PD-L1 expression on the surface of tumor cells and transmit inhibitory signals to T cells after PD-L1–PD-1 binding, which results in T cell dysfunction and tumor immune escape (39). PD-L1 is reported to be expressed on tumor cells as well as immune cells within the TME, including antigen presenting cells (APCs) (mainly macrophages and dendritic cells [DCs]), activated/depleted T and B lymphocytes, regulatory T cells (Tregs), and NK cells (8, 40). According to a comprehensive review by Sun et al. (41), the regulation of PD-L1 expression and function occurs at different levels. Several inflammatory mediators, including TNF-α, IFN-γ, IL-10, IL-17, and C5a, are inducers of PD-L1 expression (42–44). The JAK/STAT, RAS/MAPK, and PTEN-PI3K/AKT pathways are involved in the control of PD-L1 gene expression through different downstream transcription factors, such as STAT1, STAT3, IRF1, IRF3, HIF-1α, MYC, JUN, BRD4, and NF-κB (45). Corresponding DNA-binding elements other than IRF3 have been described on the PD-L1 gene promoter (46–48). Other regulatory mechanisms include microRNA (e.g., miR-513, miR-34a, miR-200, and miR-570)-mediated post-transcriptional repression and the presence of soluble PD-L1 (sPD-L1) in the blood, which may compete with membrane-bound PD-L1 for binding to PD-1 to regulate cell surface PD-L1 expression (41, 49).

IFN-γ is one of the most studied inducers of PD-L1 expression in tumors, and NF-κB (a major transcription factor of inflammation and immunity) is involved in NK cell activation to regulate IFN-γ production (50). Researchers have proposed that NF-κB directly induces PD-L1 gene transcription by binding to the latter’s promoter and that it post-transcriptionally regulates PD-L1 through an indirect pathway; thus, NF-κB may be a key positive regulator of PD-L1 expression in tumors (51). In addition, the PD-L1 promoter contains a hypoxia-inducible factor 1-alpha (HIF-1α) response element (52, 53), which drives the IKK-β gene transcription through a hypoxia response element present in the promoter while supporting NF-κB pathway activation by directly inducing p65 (54, 55); NF-κB can also induce HIF-1α transcription by directly binding to the HIF-1α promoter (56). Thus, both HIF-1α and NF-κB pathways can initiate and maintain PD-L1 expression and reinforce each other through positive feedback. Thus, the possibility that these signaling pathways can regulate PD-L1 expression in NK cells must be explored. A recent study confirmed that some myeloid leukemia cell lines and acute myeloid leukemia (AML) blasts from patients can induce PD-L1 signaling in NK cells through the PI3K/AKT/NF-κB pathway (8). Notably, the expression levels of two activating antigens, CD69 and CD25, were significantly higher in PD-L1^+^ NK cells than in PD-L1^-^ NK cells, which indicates that PD-L1^+^ NK cells show an increase in antitumor cytotoxic effector function and IFN-γ-mediated CD69 expression. The study revealed that the higher the sensitivity of the target cells to NK cell cytotoxicity (due to its negative correlation with MHC-I molecule expression), the greater the directness of the cell-cell contact between NK cells and target cells, the higher the expression of PD-L1, and the stronger the activation of CD69 NK cells (8). Therefore, PD-L1 expression on NK cells may serve as an *in vivo* biomarker for sensitivity to NK cell lysis in patients with tumors. Nonetheless, the expression and function of PD-L1 on NK cells and the involvement of PD-L1^+^ NK cells in anti-PD-L1 mAb therapy has not been comprehensively explored. An improvement in the understanding of PD-L1 expression on NK cells and signaling regulatory mechanisms is essential to understand tumor and immune cell biological characteristics and to develop NK cell-based antitumor immunotherapy.

## 4 Effects of Anti-PD-1/PD-L1 Antibodies on NK Cell Function and Corresponding Regulatory Mechanisms

Existing literature has shown the potential benefits of anti-PD-1/PD-L1 therapy in rescuing and/or improving NK cell function and improving antitumor immune responses (28, 29, 33, 57). Researchers have found that disrupting the PD-1/PD-L1 interaction can enhance the killing effect of NK cells on tumor cells of mice with several cancers, and in some models, PD-1/PD-L1 blockers are completely ineffective when mouse NK cells are depleted (32). In humanized (Hu) NOD.Cg-Prkdc^scid^Il2rg^tm1Wjl^/SzJ (NSG) mice xenografted with dedifferentiated liposarcoma (DDLPS), abundance of hCD8^+^ T subsets, such as hCD8^+^ IFN-γ^+^, hCD8^+^PD-1^+^, and hCD8^+^Ki-67^+^ cells, and hNK subsets, such as hCD56^+^IFN-γ^+^, hCD56^+^PD-1^+^, and hCD56^+^Ki-67^+^ cells, is functionally associated with anti-PD-1 effects (58). In addition, a significant increase in the number of activated hCD56^+^NKp46^+^NKG2D^+^ NK cells was also detected, which indicates that NK cells play a pivotal role in the antitumor effect of anti-PD-1 therapy (58).

**Figure 1:**
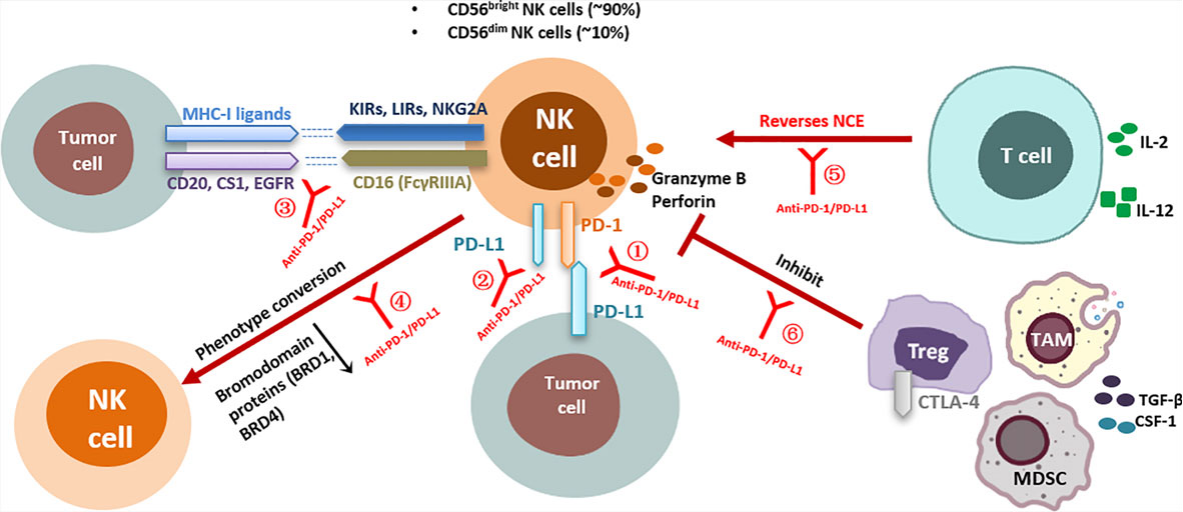
Effects of anti-PD-1/PD-L1 antibodies on NK cell function and corresponding regulatory mechanisms. Direct interaction of anti-PD-1/PD-L1 antibodies with NK cells: ①Anti-PD-1/PD-L1 antibodies block PD-1/PD-L1 inhibitory signaling in NK cells; ②Anti-PD-1/PD-L1 antibodies enhance PD-L1^+^ NK-cell antitumor activity; ③Anti-PD-1/PD-L1 antibodies enhance the ADCC effect of NK cells; ④Anti-PD-1/PD-L1 bispecific antibodies induce phenotypic transformation of NK cells; Indirect interaction of anti-PD-1/PD-L1 antibodies with NK cells: ⑤Anti-PD-1/PD-L1 antibodies indirectly reverse NK cell exhaustion by affecting CD8^+^ T cell activity; ⑥Anti-PD-1/PD-L1 antibodies relieve the inhibition of NK cell function by affecting Tregs. KIRs, killer cell immunoglobulin-like receptors; LIRs, leukocyte immunoglobulin-like receptors; NKG2A, natural killer group 2 A; CTLA-4, cytotoxicT-lymphocyte-associated protein 4; IL, interleukin; NCE, NK cell exhaustion; PD-1, programmed death receptor-1; PD-L1, programmed death-ligand 1; MHC, major histocompatibility complex; TGF-β, transformation growth factor-β; IFN-γ, interferon-γ; Tregs, regulatory T cells; CSF-1, colony-stimulating factor 1; EGFR, epidermal growth factor receptor; NK cell, natural killer cell; TAMs, tumor-associated macrophages; MDSCs, myeloid-derived suppressor cells.

PD-1/PD-L1 blocking may rescue multiple aspects of NK cell-mediated antitumor immune activity (**Figure 1**). Firstly, most studies have shown that PD-1 expression on the surface of NK cells is upregulated in the TME, and this expression plays a negative immunoregulatory role after binding to the corresponding ligands and is associated with poor tumor prognosis (29, 32, 33). PD-1/PD-L1 blockade can activate NK cells by preventing inhibitory signals between PD-1^+^ NK cells and PD-L1^+^ target cells. Secondly, the activity of NK cells is determined by a series of activation and inhibition signals, and PD-1 blockade *via* antibodies activates some positive regulatory signaling pathways or prevents other intracellular inhibitory signals (8); furthermore, the unique ADCC effect of NK cells may be activated and enhanced by some PD-1/PD-L1 blockers, and this effect triggers strong antitumor activity (59). In addition, anti-PD-1/PD-L1 therapy may indirectly affect NK cell function through other immune cells in the TME (60, 61). NK cells may, in turn, enhance tumor immune responsiveness to PD-1 antibodies by affecting other immune factors in the TME. These mechanisms are reviewed in detail below.

### 4.1 Direct Interaction of Anti-PD-1/PD-L1 Antibodies With NK Cells

#### 4.1.1 Anti-PD-1/PD-L1 Antibodies Block PD-1/PD-L1 Inhibitory Signaling in NK Cells

As discussed above, the binding of PD-L1 expressed by tumor cell surface to PD-1 expressed by activated NK cells potentially inhibits NK cell-dependent immune surveillance and mediates antitumor immune responses. However, studies on the mechanism by which PD-1 inhibits NK cell response against tumors and the possibility of inducing NK cell responses *via* PD-1/PD-L1 blockade are still scarce. In one study, CD16- or ILCs-activated NK cells showed upregulated expression of PD-1, as well as CD69, CD107a, IFN-γ, and granzyme B; however, the expression of these molecules was downregulated and that of CD16 surface density was significantly downregulated after the binding of PD-1 to PD-L1 (33). PD-1 blockade increased NK cell activation and cytotoxicity, but only in patients with HNCs showing high PD-L1 expression (33). Another study used anti-PD-1/PD-L1 antibodies to block the interaction between PD-1and PD-L1 and found that this strategy could help reverse PD-1 inhibition and the dysfunctional state of PD-1^+^ NK cells. This blockade leads to a significant increase in NK cell cytotoxicity and cytokine production, inhibition of tumor growth *in vivo*, and obvious improvement in NK cell-based antitumor response (32). Similarly, a previous study found that blocking PD-1/PD-L1 signaling on the surface of NK cells significantly enhanced IFN-γ production, CD107a expression, and cell degranulation, and inhibited NK cell apoptosis *in vitro*. More importantly, PD-1 blocking antibody was found to significantly inhibit the growth of xenograft tumors, and NK depletion completely abolished this inhibition of tumor growth (29). However, inhibition of tumor growth could be completely abolished by NK depletion. Furthermore, PD-1 may exert its inhibitory effect on NK cells by interfering with AKT activation, and PD-1/PD-L1 blockade activates NK cells by enhancing the PI3K/AKT signaling pathway in them (29). In addition, anti-PD-1/PD-L1 antibodies can enhance the secretion of tumor necrosis factor-related apoptosis-inducing ligand (TRAIL) by inhibiting PD-1 in NK cells, effectively improving the antitumor activity of these cells (62). IFN-β has been shown to activate NK cells and induce cytotoxicity against tumor cells by upregulating NK cell-surface membrane-bound and soluble TRAIL expression, which leads to subsequent activation of the TRAIL receptor signaling pathway and apoptosis in nasopharyngeal carcinoma (NPC) cells (62). Subsequently, blocking the PD-1/PD-L1 checkpoint in the presence of IFN-β further increased the killing activity of NK cells against NPC cells, and this suggests that blocking PD-1 in activated NK cells may increase the secretion of soluble TRAIL and contribute to the killing of TRAIL NPC cells (62). This study revealed a new mechanism by which IFN-β and anti-PD-1 antibodies enhance the antitumor effect of NK cells and provide ideas for the therapeutic strategy of the combination of IFN-β and anti-PD-1.

Recently, researchers have found that repetitive irradiation can increase PD-L1 levels in non-small cell lung carcinoma (NSCLC) cells while reducing NKG2D ligand levels, which may reduce the sensitivity of lung tumor cells to the cytotoxic effect of NK cells and the possibility that tumor cells escape immune responses (63). Mechanistic studies revealed that IL-6-MEK/ERK signaling contributes most significantly to the upregulation of PD-L1 or downregulation of NKG2D ligand in radioresistant cells (63), while in another similar study, IL‐6‐JAK/STAT3 signaling was shown to contribute significantly to this process (64). Subsequently, researchers examined PD‐1 levels in NK cells and found that PD‐1 expression could not be detected in primary NK cells, but this expression increased when NK cells were exposed to tumor cells; in other words, there was a PD‐L1/PD‐1 interaction between tumor cells and NK cells, which inhibited the activity of NK cells (63). When neutralizing antibodies against PD-L1 were added to radioresistant cell/NK cell co-cultures, this resistance was reduced and the susceptibility of tumor cells to NK cell cytotoxicity increased, presumably because PD‐L1/PD‐1 interaction was blocked (63). In addition, the added PD-L1 antibodies effectively reversed NK cell activity by releasing PD-1 from the PD-L1–PD-1 complex; these antibodies also effectively reversed the expression of NKG2D ligand on tumor cells, which further enhanced the killing ability of NK cells against tumor cells (63). In summary, the PD-1/PD-L1 axis is an inhibitory/regulatory signal for the interaction between tumor cells and NK cells, and blocking of PD-1/PD-L1 interaction may be an effective antitumor immunotherapeutic strategy that is based on the reversal of NK cell dysfunction.

#### 4.1.2 Anti-PD-1/PD-L1 Antibodies Enhance PD-L1^+^ NK-Cell Antitumor Activity

Anti-PD-1/L1 mAbs may act on NK cells through other non-PD-1 dependent pathways in addition to the PD-1/PD-L1 pathway. Upon encountering and being activated by NK-susceptible tumor cells, NK cells not only secrete cytokines and cytolytic granules, but also upregulate the expression of PD-L1 on their own surface through the PI3K/AKT/NF-κB pathway (8). This population of PD-L1^+^ NK cells is essential for the antitumor activity of PD-L1 mAbs. Upon application of atezolizumab, an mAb medication against PD-L1, PD-L1^+^ NK cells express significantly increased levels of granzyme B, IFN-γ, and CD107a, which results in a significant increase in antitumor activity and ultimately a significant decrease in tumor burden in mice (8). Furthermore, it was confirmed that anti-PD-L1 mAbs directly activate PD-L1^+^ NK cells through the p38/NF-κB pathway in PD-L1^−^ tumors (8). Upregulation of PD-L1 by NK cells, as well as the direct effect of atezolizumab on NK cells, leads to NF-κB activation, which creates a positive feedback loop in the presence of excessive immunotherapeutic agents to consistently induce PD-L1 expression and further activate NK cells (8). In the loop, the binding of anti-PD-L1 mAbs to PD-L1 upregulates PD-L1 expression on the surface of NK cells, thus, increasing the number of binding sites for the drug; this, in turn, leads to sustained activation of p38, which further transmits strong activation signals to NK cells to maintain their cytotoxic and cytokine secretion characteristics (8). The study suggested that anti-PD-L1 mAb therapy has a unique therapeutic effect against PD-L1-negative tumors (8). Based on the fact that PD-L1^+^ NK cells act through a PD-1-independent pathway, the discovery of new antitumor mechanisms provides insights into the activation of NK cells and a potential explanation for the response to anti-PD-L1 mAb therapy in some patients who lack PD-L1 expression.

#### 4.1.3 Anti-PD-1/PD-L1 Antibodies Enhance the ADCC Effect of NK Cells

Most PD-1/PD-L1 inhibitors (including nivolumab, pembrolizumab, etc.) have human IgG4 which has low fragment crystallizable (Fc) effector activity. These would be expected to have low ADCC. Atezolizumab is aglycosylated and would be expected to have no ADCC. These all prevent potent ADCC against non-tumor cells expressing PD-L1. However, avelumab, a fully human IgG1 anti-PD-L1 mAb containing a wild-type Fc that induces ADCC (65), has shown toxicity and efficacy similar to those of Fc-modified anti-PD-1/PD-L1 mAbs in several phase I and II clinical trials (66). FcγRIIIA expressed on NK cells can activate NK cell-induced cytotoxicity by recognizing the Fc portion of tumor-bound antibodies and releasing cytotoxic factors and cytokines that recruit and activate other immune cells with specific antitumor activity in the presence of tumor antigen-targeting antibodies (16). Investigators have demonstrated that avelumab triggers and enhances NK cell-mediated ADCC against TNBC cells expressing a certain level of PD-L1, which results in a significant increase in tumor cell lysis. Park et al. (59) also demonstrated that when NK cells were co-cultured with wild-type Fc anti-PD-L1 mAbs, which can induce ADCC effects on NK cells, the cytotoxicity against PD-L1-positive tumor cell lines was significantly enhanced. Therefore, some anti-PD-L1 mAbs may act as both tumor antigen-targeting antibodies and anti-PD-L1 inhibitors, which leads to the activation of NK cell and CD8^+^ T cell and improvement in therapeutic efficacy.

The magnitude of the ADCC effect of NK cells evoked by anti-PD-L1 mAbs may be modulated by other factors. First, polymorphisms in FcγRIIIA may affect NK cell-mediated interindividual variability in the ADCC effect (67). Although this association was identified in studies involving different tumor antigen-targeting mAbs of the IgG1 isotype (rituximab, trastuzumab, and cetuximab), it may not be generalizable to different settings (68). Preliminary *in vitro* results showed that avelumab-mediated ADCC was more effective in patients with NK cells expressing the high-affinity CD16 valine (V) allele than in those with NK cells expressing the low-affinity phenylalanine (F) allele and F/F genotype (65); however, this difference in effectiveness needs to be verified in clinical studies. Second, the release of some cytokines, such as IL-2 and IL-15, as well as subsequently stimulated IFN-γ, can stimulate the enhancement of avelumab-triggered cytokine production and degranulation in NK cells while increasing lytic activity against tumor cells (67). In addition, in a previous study, investigators enhanced the ADCC of avelumab against many types of cancer cells through epigenetic priming of NK cells and tumors (69). This evidence suggests that therapeutic strategies that block PD-1/PD-L1 while inducing the ADCC action of NK cells may enhance existing immunotherapeutic efficacy. Considering that the magnitude of NK cell-based ADCC effects induced by anti-PD-L1 mAbs can be regulated by some factors such as immunomodulators (IL-15 or IL-2), combining drugs targeting these factors (67) may improve the effectiveness of immunotherapeutic strategy.

#### 4.1.4 Anti-PD-1/PD-L1 Bispecific Antibodies Induce Phenotypic Transformation of NK Cells

Bromodomain proteins, such as BRD1 and BRD4, play a role in the development of immune and hematological cells and in the regulation of tumor inflammation (70–72). In a previous study on high-grade serous ovarian cancer (HGSC), BRD1 expression was found to be low in tumor cells and high in immune cells, which is associated with significant downregulation of T cell- and NK cell-surface activity markers (GZMA, GZMB, IFNG, and NKG7) and upregulation of the naïve T cell marker TCF7 (73). This study performed immune function and single-cell RNA-seq transcriptional profiling of novel HGSC organoid/immune cell co-cultures treated with unique bispecific anti-PD-1/PD-L1 antibodies versus monospecific anti-PD-1 or anti-PD-L1 antibodies (control) (73). It revealed that bispecific antibodies uniquely induced a transition from inert to more active and cytotoxic NK cell phenotypes and a transition from naïve to more active and cytotoxic progenitor-exhausted phenotypes of CD8^+^ T cells after treatment. It was further found that these superior cell state changes were driven in part by the downregulation of BRD1 expression induced by bispecific antibodies in immune cells (73). The inhibitory effect of the small molecule inhibitor, BAY-299, on BRD1 partially leads to increased NK cell maturation, activation, and tumor cell killing *via* alteration of the chromatin pathway of key immune transcription factors (e.g., GATA3, TBX21, and TBXT), induces similar state transitions in immune cells *in vitro* and *in vivo* and demonstrates the *in vivo* efficacy of bispecific antibodies (73). Therefore, changes in the activity and cytotoxic status of NK cells and T cells may be the key to driving the induction of effective antitumor immune responses using bispecific antibodies, by partially eliminating some TME-driven dysfunction through epigenetic changes (BRD1 downregulation or inhibition).

### 4.2 Indirect Interaction of Anti-PD-1/PD-L1 Antibodies With NK Cells

#### 4.2.1 Anti-PD-1/PD-L1 Antibodies Indirectly Reverse NK Cell Exhaustion by Affecting T Cell Activity

Regulative effects of NK and CD8^+^ T cells on each other have been reported in many infection models (74, 75) and antigen-independent IL-2 models (76), and PD-1/PD-L1 inhibitors may indirectly alter NK cell function by affecting CD8^+^ T cell activity. NK cell exhaustion (NCE) has been identified as a self-regulatory mechanism responsible for inducing dysfunctional phenotypes to prevent exacerbated immune responses under conditions of chronic stimulation (77), and it is a crucial mechanism involved in tumor or viral evasion of immune responses. IL-2 is a pleiotropic cytokine that activates T cells, NK cells, and dendritic cells. IL-2 binds to IL-2 receptors (IL-2Rs), including CD32 (IL-2Rγc), CD122 (IL-2Rβ), and CD25 (IL-2Rα). IL-2Rs showed different affinities for cytokines, with CD25 (IL-2Rα) having the highest IL-2 affinity. CD25 is constitutively expressed on Tregs; therefore, IL-2 treatment can lead to the expansion of these cells, thus mediating NK and CD8^+^ T cell suppression through multiple mechanisms (76, 78, 79). A recent study evaluated the role of the PD-1/PD-L1 pathway in NK cell activation, function, and depletion in a mouse model of IL-2-dependent depletion (80) and found that anti-PD-1 therapy provides an activating advantage to CD8^+^ T cells in the competition for IL-2 by promoting CD8^+^ T cell expansion, activation, and functional phenotype; consequently, the quantity of stimulatory cytokines available to NK cells becomes limited, which results in a delayed NCE, improved NK cell activation, higher proliferative capacity, and enhanced granzyme B production. This evidence suggests that the phenotypic and functional benefits observed after the chronic stimulation of NK cells by anti-PD-1 therapy are indirectly mediated by the effect of anti-PD-1 antibodies on CD8^+^ T cells, rather than by the direct effect of anti-PD-1 drugs on NK cells; this can be demonstrated by the reversal of the benefit of anti-PD-1 therapy on NK cells by the depletion of CD8^+^ T cells. Disruption of the balance between NK and CD8^+^ T cells affects the homeostatic response to each other’s stimuli, and this resource competition (i.e., IL-2) delays the onset of NCE. Thus, there is a delicate balance between CD8^+^ T cells, Tregs, and NK cells that is partly regulated by their ability to respond to cytokines. Achieving a balance between these immune cells may be important for achieving long-term efficacy of immunotherapy. Although dual regulation between CD8^+^ T cells and NK cells is caused by competition for space and resources, direct or indirect lysis, and functional inhibition, these two populations can also work together to mount a stronger response. Another study found that dual blockade of PD-1 and IL-10 enhanced cytokine secretion, NK degranulation, and killing target cell function of NK cells by restoring HIV-specific CD4^+^ T cell function, thus establishing a previously unappreciated relationship between CD4^+^ T cell injury and NK cell depletion in HIV infection (81). This important evidence also fully suggests that PD-1 blockade may enhance immunotherapy by improving collaboration between CD4^+^ T cells and NK cells.

#### 4.2.2 Anti-PD-1/PD-L1 Antibodies Relieve the Inhibition of NK Cell Function by Affecting Tregs

Immunosuppressive cells such as tumor-associated macrophages (TAMs), myeloid-derived suppressor cells (MDSCs), and Tregs in the TME inhibit NK cell proliferation, infiltration, and activation by secreting immunosuppressive cytokines (TGF-β and IL-10) or by interfering with NK cell receptor expression and activation; thus, these cells facilitate tumor immune escape and promote progression and metastasis (82, 83). It can, therefore, be hypothesized that PD-1/PD-L1 blockade prevents the induction of immunosuppression and improves NK cell efficacy to increase the survival of a tumor-bearing animal. A previous study showed that anti-PD-L1 therapy has no direct effect on the cytotoxicity or cytokine secretion of PD-1-negative PM21 particle-expanded NK cells in response to PD-L1^+^ targets *in vitro*; however, secretion of a large quantity of IFN-γ by NK cells significantly improved antitumor efficacy, and long-lasting retention of their cytotoxic phenotype by NK cells can be observed *in vivo* (84). Thus, the investigators continued to explore the specific mechanism by which anti-PD-L1 therapy enhances the antitumor efficacy of NK cells. They found that PM21-NK cells are highly cytotoxic to tumor cells and secrete IFN-γ upon stimulation, which leads to the induction of PD-L1 expression in tumors and consequently to the induction and *in situ* proliferation of Tregs in the TME (84). Subsequently, blockade of PD-L1 mitigated the induction of Treg expansion and associated immunosuppressive responses, which in turn improved the NK cell phenotype and cytotoxic activity, as well as survival in treated animals (84). The number of CD57^+^ NK cells has increased, a population with high cytotoxic capacity and responsiveness *via* CD16 binding, and the presence of these cells is correlated with better outcomes in patients with squamous cell lung cancer in other reports (85, 86). The expansion of Tregs may occur in the TME with PD-L1 expression, and Tregs have been reported to suppress the survival and cytotoxic function of NK cells through several mechanisms, including TGF-β surface presentation (87–89). Thus, anti-PD-L1 therapy may improve NK cell antitumor efficacy by reducing *in situ* Treg generation and preventing Treg induction.

## 5 NK Cells Enhance Anti-PD-1/PD-L1 Antibody Efficacy by Affecting Other Immune Cells in the TME

NK cells and other immune cells in the TME mutually regulate each other; for example, PD-L1^+^ liver-resident NK (LrNK) cells have an inhibitory interaction with PD-1^+^ T cells (90). NK cells can indirectly affect the antitumor immune function by acting on these immune cells, thereby enhancing the response of tumors to anti-PD-1/PD-L1 antibodies. The large number of beneficial effects of tumor-infiltrating NK cells can be mediated by modulating the TME and promoting T cell recruitment and activation. An earlier study that used mixed lymphocyte cultures showed that NK cells are required for the differentiation and activation of CD8^+^ T cells with cytotoxic functions (91). Another study that used *in vitro* co-culture and *in vivo* xenograft adoptive transfer experiments demonstrated that high-quality NK cells (iNK) derived from induced pluripotent stem cells (iPSCs) can recruit and activate T cells, allow them to respond to PD-1 blockade, and enhance inflammatory cytokine production and tumor elimination (92). NK cells in tumor-infiltrating and draining lymph nodes were reported to show upregulation of the inhibitory molecule PD-L1, which transmits an inhibitory signal by interacting with PD-1 expressed on DCs to limit the activation of these cells; this in turn leads to a decrease in the priming ability and memory response of tumor-specific CD8^+^ T cells (61). When blocking the interaction between NK cells and DCs, a significantly higher frequency of CD8^+^ T cells, level of IFN-γ production, and capacity for cytotoxicity were observed (61). In this model, tumor cells induced the modulation of DC activation *via* PD-L1^hi^NK cells, which reduced the priming capacity of CD8^+^ T cells. Conversely, PD-1/PD-L1 inhibitors may reverse the inhibitory effect of PD-1-PD-L1 interaction on CD8^+^ T cells by disrupting the direct interaction pathway between NK cells and DC cells; this presumably activates NK cell activity and needs to be explored further in future studies. In addition to interacting with DCs through the PD-1/PD-L1 axis, a study has shown in human melanoma that NK cells stably form conjugates with stimulatory dendritic cells (SDCs) in mouse TME and positively regulate the abundance of SDCs in tumors by producing FLT3LG, the cDC1 formative cytokine. SDCs are important in stimulating cytotoxic T cells and driving anticancer immune responses (60). Although anti-PD-1 immunotherapy for cancer primarily targets T cells, NK cell frequencies correlate with protective SDCs in human cancers, with patient responsiveness to anti-PD-1 immunotherapy, and with prolonged overall survival (60).

In summary, blocking PD-1/PD-L1 may activate the systemic immune response by reversing the inhibitory effect of NK cells on other immune cells or activating the antitumor capability of other immune cells by NK cells, which is an effective antitumor immunotherapy strategy. Therefore, a combination therapy based on NK cells and anti-PD-1/PD-L1 drugs can be established.

## 6 Combination Therapy of NK Cells and Immune Checkpoint Inhibitor

Considering NK cell-mediated cytotoxicity does not require MHC class I and the unique effects of anti-PD-1/PD-L1 mAb therapy on NK cell function described above, adoptive transfer of autologous or allogeneic NK cells together with anti-PD-1/PD-L1 antibodies may potentially enhance the outcomes of patients receiving cancer immunotherapy. A previous study explored the effects of a combination of NK cells with PD-L1 blockers, regardless of PD-1 expression on NK cells or the initial PD-L1 status of the tumor cells (84). The OS of animals treated with a combination of anti-PD-L1 antibodies and PM21-NK cells was twice as higher than that of those treated with anti-PD-L1 antibodies alone (48 days vs. 24 days, *P* = 0.0001) (84). The first clinical study on the combination of anti-PD-1 antibodies (pembrolizumab) and allogeneic NK cells in patients with advanced NSCLC in China was recently published (93), and it reported the doubling of the number of NK cells, significant increases in the levels of cytokines such as IL-2, TNF-β, and IFN-γ, and significant decreases in the levels of multiple tumor markers such as circulating tumor cells (CTCs) after treatment in the combination therapy group. Patients in the combined therapy group had a significantly better overall response rate (36.5% vs. 18.5%) and a significantly better survival outcome (OS: 15.5 months vs. 13.3 months; PFS: 6.5 months vs. 4.3 months; all *P* < 0.05) than did those in the anti-PD-1 antibody alone group; moreover, the benefits were more significant in the combination therapy group [tumor proportion score [TPS] ≥ 50%] than in the latter group. In addition, patients who received multiple courses of NK cell infusion showed a better OS than those who received a single course of NK cell infusion (18.5 months vs. 13.5 months) (93). The treatment was well tolerated throughout the treatment. All adverse events were below grade 4, with grade 2 events comprising the majority of events. All symptoms were relieved after symptomatic treatment. Therefore, a combination therapy of anti-PD-L1 antibodies and NK cells can significantly enhance the antitumor effect, improve the survival benefit, and serve as new treatment regimen for previously treated patients with advanced PD-L1^+^ NSCLC. In addition, the combination of anti-PD-1/PD-L1 antibody therapy and drugs that enhance the antitumor effects of NK cells through other mechanisms, including a combination of anti-NKG2A mAb (monalizumab) (94), KIR blockade (95), cytokine therapy (96), and therapies targeting other checkpoint receptors such as CTLA-4, LAG-3, CD96, and TIGIT, may have a synergistic effect (97–99); therefore, this strategy may be beneficial for improving the efficacy of immunotherapy and overcoming drug resistance.

## 7 Summary and Prospects

The use of anti-PD-1/PD-L1 antibodies could be a promising option for the successful treatment of malignancies as they help overcome T-cell depletion by blocking the PD-1/PD-L1 signaling pathway in the TME. However, most patients and tumor types have shown low/no response to these therapies, or some patients with predicted no or low response to treatment have shown significant benefit; therefore, in-depth exploration of the mechanism of action and efficacy of ICIs is necessary to effectively screen for suitable patients to expand the benefits to them. Accumulating evidence suggests that immune cells other than T cells, such as NK cells, are also involved and play an important role in the PD-1/PD-L1 blocking process; these non-T cells have become an effective complement to T cell immune responses because of their unique advantages, especially against MHC-deficient tumors that show low antigenicity or are resistant to T cell recognition and cytolysis. At present, findings of studies on the expression of PD-1 and PD-L1 on NK cells and their functions are not completely consistent, and further evidence derived from human NK cell-based research is necessary. According to our review, PD-1/PD-L1 antibodies can rescue NK cell from multiple aspects of dysfunction caused by TME, revitalize the cytotoxic activity of these cells against tumors, and further initiate and enhance T cell-mediated adaptive antitumor immunity; NK cells can also indirectly enhance the efficacy of PD-1/PD-L1 blockade by affecting other immune cells in TME. On the basis of these findings, multiple combination strategies, such as the use of a combination of PD-1/PD-L1 inhibitors with drugs that promote NK cell infiltration, persistence, and activation in tumors (e.g.,cytokines, stimulator of IFN gene [STING] agonists) and resistance to inhibitory TMEs (e.g., anti-TGF-β mAbs), are under investigation in basis research studies or clinical trials; these strategies aim to further increase the antitumor activity of NK cells and improve the tumor response to immunotherapy. At present, our understanding of the role of NK cells during PD-1/PD-L1 blockade is still insufficient and needs to be further explored and validated using basic mechanistic studies. A comprehensive and in-depth understanding of the response mechanism of PD-1/PD-L1-based immunotherapy and the interaction mechanism between each immune cell in TME with another or with tumors can lay a foundation for optimizing the efficacy of existing treatments, developing new immunotherapies based on NK cells, and developing combination therapy strategies.

## Author Contributions

All authors listed have made a substantial, direct, and intellectual contribution to the work and approved it for publication.

## Funding

This work was supported by grants from Jilin Provincial Department of Science and Technology Project (20190303146SF); Jilin Provincial Department of Finance Project (JLSWSRCZX2020-0023); Jilin Province Biotherapeutic Science and Technology Innovation Center Project (20200602032ZP).

## Conflict of Interest

The authors declare that the research was conducted in the absence of any commercial or financial relationships that could be construed as a potential conflict of interest.

## Publisher’s Note

All claims expressed in this article are solely those of the authors and do not necessarily represent those of their affiliated organizations, or those of the publisher, the editors and the reviewers. Any product that may be evaluated in this article, or claim that may be made by its manufacturer, is not guaranteed or endorsed by the publisher.
